# FLEXIBILITY IN PRACTICE: CASE EXAMPLES OF THE ROLE OF A BRAIN HEALTH INTERVENTIONIST IN THE AGEWISE-AP PROGRAM

**DOI:** 10.1093/geroni/igae098.4357

**Published:** 2024-12-31

**Authors:** Jaye McLaren, Lauren Moo, Andrew Nguyen, Maureen O’Connor

**Affiliations:** Department of Veterans Affairs, Bedford, Massachusetts, United States; Department of Veterans Affairs, Bedford, Massachusetts, United States; Department of Veterans Affairs, Bedford, Massachusetts, United States; Department of Veterans Affairs, Bedford, Massachusetts, United States

## Abstract

Healthy brain aging and reducing the risk of dementia is important to older adults. The Aging Well through Interaction and Scientific Education program (AgeWISE) provides group education (12 sessions) on lifestyle factors and techniques to manage age-related cognitive change. Participants who completed AgeWISE reported feeling that they had more control over their brain health, but many requested additional supports to help them make lifestyle changes important to them. We added an 8-session one-on-one action plan component to the AgeWISE program (AgeWISE-AP) to help participants make personalized lifestyle modifications that support healthy brain aging. AgeWISE-AP utilizes a Brain Health Interventionist (BHI) to guide participants in the creation and implementation of individualized, client-centered goals while adhering to a manualized scaffolding. Fifteen participants have engaged in AgeWISE-AP to date. We will provide case examples illustrating the flexibility required from the BHI to manage individual needs in goal setting, adapt goals as needed, and successfully guide participants in behavior change within a manualized program. The AgeWISE-AP program requires the BHI to have strong clinical skills and content-specific training to support goal setting and implementation that is client-centered while also ensuring the sessions are operating within the structure of the manualized program. Walking this fine line is imperative for the success of the program as well as for its replicability in other settings.

